# Key Synchronization Method Based on Negative Databases and Physical Channel State Characteristics of Wireless Sensor Network

**DOI:** 10.3390/s24196217

**Published:** 2024-09-25

**Authors:** Haoyang Pu, Wen Chen, Hongchao Wang, Shenghong Bao

**Affiliations:** School of Cyber Science and Engineering, Sichuan University, Chengdu 610207, China; puhaoyang1@stu.scu.edu.cn (H.P.); wanghc@stu.scu.edu.cn (H.W.); heitan@stu.scu.edu.cn (S.B.)

**Keywords:** wireless sensor network, synchronous key distribution, negative database, Channel State Information, data security transmission

## Abstract

Due to their inherent openness, wireless sensor networks (WSNs) are vulnerable to eavesdropping attacks. Addressing the issue of secure Internet Key Exchange (IKE) in the absence of reliable third parties like CA/PKI (Certificate Authority/Public Key Infrastructure) in WSNs, a novel key synchronization method named NDPCS-KS is proposed in the paper. Firstly, through an initial negotiation process, both ends of the main channels generate the same initial key seeds using the Channel State Information (CSI). Subsequently, negotiation keys and a negative database (NDB) are synchronously generated at the two ends based on the initial key seeds. Then, in a second-negotiation process, the NDB is employed to filter the negotiation keys to obtain the keys for encryption. NDPCS-KS reduced the risk of information leakage, since the keys are not directly transmitted over the network, and the eavesdroppers cannot acquire the initial key seeds because of the physical isolation of their eavesdropping channels and the main channels. Furthermore, due to the NP-hard problem of reversing the NDB, even if an attacker obtains the NDB, deducing the initial key seeds is computationally infeasible. Therefore, it becomes exceedingly difficult for attackers to generate legitimate encryption keys without the NDB or initial key seeds. Moreover, a lightweight anti-replay and identity verification mechanism is designed to deal with replay attacks or forgery attacks. Experimental results show that NDPCS-KS has less time overhead and stronger randomness in key generation compared with other methods, and it can effectively counter replay, forgery, and tampering attacks.

## 1. Introduction

The Internet of Things (IoT) has been widely applied in people’s daily lives. It finds applications in both civilian domains—such as e-commerce, smart homes, smart cities, and healthcare [1,2,3,4,5]—and military fields, such as military reconnaissance [6]. Among the enormous IoT devices, wireless sensor networks (WSNs) are commonly utilized due to their advantages of low cost, reliability, scalability, ease of deployment, etc. Since WSNs may be deployed in untrustworthy environments, they are very susceptible to network attacks. Therefore, data secure transmission in WSNs attracted lots of attention from researchers [7]. Secure transmission in WSNs involves ensuring data confidentiality, integrity, and reliability. Researchers have proposed various methods to ensure the security of data transmission in WSNs, including traditional encryption algorithms, lightweight block cipher algorithms, and physical layer security [8]. The communication key remains the most crucial security parameter in all these methods. However, traditional Internet Key Exchange (IKE) typically requires a pre-distribution of keys or relies on centralized key servers, such as CA/PKI (Certificate Authority/Public Key Infrastructure). In WSNs, many sensor nodes dynamically join or leave the network, and the network topology constantly changes. A centralized key server may become a bottleneck or a target for attackers, leading to a single point of failure. Therefore, relying on CA/PKI for key distribution in WSNs is challenging. Although pre-distributing fixed keys avoids the risk of key leakage during transmission, this method lacks flexibility and security: using the same key for data encryption over a long period still increases the risk of key compromise by attackers. Furthermore, key generation algorithms based on computational hardness pose efficiency and cost issues for WSNs with limited computing capabilities. Thus, there is a need for a novel key synchronization method suitable for WSNs, allowing sensor nodes to autonomously and securely synchronize communication keys without exchanging critical negotiation information through the open network.

Consequently, this paper proposes a novel Negative Database and Physical Channel State Key Synchronization (NDPCS-KS) for WSNs. NDPCS-KS exploits the physical isolation of channels between sensor nodes to synchronously generate initial key seeds at both ends of the communications through Channel State Information (CSI) in the initial-negotiation phase. Based on the initial key seeds, negotiation keys and negative databases (NDBs) are generated. Then, the NDBs are used to conduct a second negotiation, which filters the negotiation keys to obtain the final communication keys. The entire negotiation process involves both initial-negotiation and second negotiation, which is referred to as dual negotiation.

Additionally, a lightweight anti-replay and identity verification mechanism is introduced to prevent replay attacks and counter against forgery and tampering. NDPCS-KS operates without the need for reliable third parties such as CA/PKI, using hash tokens maintained among sensor nodes to prevent replay, and it computes hash values for verifying identities, ensuring the integrity and reliability of data transmission. The main contributions of this paper are outlined as follows:

1. Avoidance of Confidential Information Leakage: NDPCS-KS avoids reliance on Certificate Authorities (CAs) or Public Key Infrastructure (PKI), eliminating the need for pre-distributed keys or dependence on centralized key servers. It dynamically generates keys based on the current state of the physical channels, ensuring no confidential information is transmitted over the network in the key exchange process, preventing the leakage of secrets.
2. Security key exchange mechanism: By leveraging the isolation of the physical channels, NDPCS-KS prevents attackers from eavesdropping on the main channel’s state information to obtain initial key seeds. For the first time, the NDB is employed in the IKE process of WSNs to generate encryption keys. The irreversible nature of the NDB prevents the attackers from generating legitimate keys.
3. Resistance to Attacks: NDPCS-KS resists replay attacks, identity forgery, and data packet tampering, ensuring data integrity and reliability. Compared to traditional key exchange mechanisms, it offers higher security and flexibility.
4. Extensive Experimental Validation: Extensive experiments have been conducted to compare the effectiveness and feasibility of NDPCS-KS with other methods, demonstrating its effective performance and capability to resist various attacks.

The organization of this paper is as follows: Section 2 introduces related work, the current state of research on key generation in WSNs, the current state of research on key negotiation and quantization, and the concept and research status of the NDB; Section 3 introduces the system model; Section 4 introduces the key technologies and algorithms; Section 5 analyzes the security of NDPCS-KS; Section 6 validates the effectiveness and security of NDPCS-KS through experiments; Section 7 concludes the paper.

## 2. Related Work

### 2.1. Current State of Research on Key Generation in WSNs

For the security of the IKE process in WSNs, researchers have initially focused on pre-distribution key schemes. The basic idea involves distributing shared keys between nodes and base stations before network deployment. Dai et al. [9] proposed a matrix-based key pre-distribution scheme for secure communication in WSNs, utilizing the Lower–Upper matrix method for efficient key establishment. This method ensures reliable connectivity and enhances resilience against node capture by adopting polynomial-based key pre-distribution. Kuang [10] improved upon the basic random key pre-distribution scheme by enhancing network connectivity and security performance. The scheme introduces a “neighbor” node expansion protocol to protect isolated nodes and establish secure paths, thus increasing the global connectivity and security of the network. Yin et al. [11] proposed a multipath key establishment mechanism based on secret sharing to counteract attacks in WSNs. This mechanism can be integrated with other pre-distribution schemes to protect the network from various attacks, including stop-forwarding attacks and key leakage through intermediate nodes. Pre-distribution key methods face issues with poor flexibility and scalability. In larger networks, pre-distribution schemes can lead to key distribution and management becoming bottlenecks and increase the system’s vulnerability.

Researchers have proposed dynamic key distribution methods to address the limitations of pre-distribution schemes. These methods dynamically generate temporary keys, thus overcoming the flaws of static pre-allocated key schemes. Shu et al. [12] introduced a dynamic nonlinear key distribution method to reduce the number of keys shared between nodes. They utilize a seed key to encrypt and distribute keys, deriving data keys from the distributed keys and using the data keys for data encryption. They achieved confidential communication in WSNs through dynamic nonlinear key distribution with minimal shared seed keys. Chen et al. [13] developed a dynamic key management scheme that uses a shared seed key, node identifiers, packet counts, and random numbers to generate dynamic distribution keys. It generates a distribution key in a single communication packet and uses it only once. Chen also proposed key management principles for generating distribution keys and introduced a confidentiality algorithm. It showed that the dynamic key management scheme is secure and feasible, enhancing network security as keys can be immediately replaced after data transmission, reducing the risk of key leakage. However, dynamic key distribution methods also face challenges, such as the frequency of key updates, distribution efficiency, and ensuring timely key updates without adding excessive communication load. Yousefpoor and Barati [14] proposed an intelligent dynamic key management system designed for WSNs, employing fuzzy logic for path key generation and adding new nodes to the network. The system adopts a hierarchical network approach and leverages pre-allocation and post-deployment mechanisms for key distribution. The simulation results of this system indicated that compared with other key management systems, it is more efficient regarding communication load, storage space, and energy consumption.

In response to the limitations of dynamic key distribution methods, recent studies have proposed key generation methods based on wireless channels. These methods utilize the physical layer characteristics of wireless communications, such as signal strength or the randomness of channel responses, to generate keys. The advantage of this approach is that it does not rely on traditional key distribution protocols but instead exploits the inherent properties of the wireless channel itself. This enhances the security of key generation, as the isolation features of the physical channels make it difficult for external attackers to eavesdrop on the channel state. Xiong et al. [15] introduced a new device-to-device key generation strategy involving multiple randomnesses and a cooperative communication mechanism assisted by relay nodes. It allows Alice and Bob to acquire correlated information and generate keys by alternately transmitting random signals during each coherent period, leveraging the randomness and reciprocity of the wireless propagation channel. Altun et al. [16] present a secret key generation method for WSNs by using physical layer features. The method addresses latency and bandwidth issues in dense networks by exploiting the superposition property of wireless channels. It also provides security against eavesdroppers by using simultaneous transmissions in the same time and frequency block, ensuring scalability and secure communication in highly populated networks. Wei and Saha [17] proposed KNEW, which is a novel method that extracts implicit features from reciprocal channels to derive keys with high consistency. KNEW simultaneously trains two neural networks to map each other’s channel estimation into different domains, namely latent spaces, which remain inaccessible to adversaries. The model also minimizes the distance between the latent spaces generated by the two trusted nodes, improving the key inconsistency rate. Rangarajan [18] proposed a sensor node security enhancement method in WSNs based on an improved Diffie–Hellman key exchange, enhancing security by generating hashes for the payload transmitted over the network; thus, it can defend against man-in-the-middle attacks. Moara-Nkwe et al. [19] propose a physical layer secure communication scheme tailored for static and low-latency industrial IoT environments. The key contributions include the introduction of a multi-antenna controller and single-antenna sensor communication model, analysis of passive eavesdropping and active attack threats, and the development of a Random Pilots (RPs)-based key generation method. The scheme enhances security and transmission efficiency through self-interference-assisted one-time pad encryption, effectively addressing the challenges posed by stable channel conditions over time. Ji et al. [20] present a new physical layer secure key generation and refreshment scheme designed for WSNs. The process involves generating initial keys using pilot signals and channel estimation, which is followed by error correction and privacy amplification to ensure key security. The scheme introduces a key refreshment mechanism that incorporates information from all previously generated keys, forming a hash key chain to enhance long-term security. Experimental results validate the scheme’s effectiveness and security under various channel conditions, offering a practical solution for secure key management in WSNs. Aldaghri et al. [21] proposed a new method for generating secret keys in static environments by introducing randomness to address the issue of ultra-low key generation rates in traditional methods. This method, called “induced randomness”, allows two parties (Alice and Bob) to utilize the unique characteristics of the wireless channel to generate high-rate secret keys. Alice and Bob independently generate local random values and combine them with the inherent randomness of the wireless channel to create highly correlated samples for key generation. This method can be applied in both direct communication and relay-assisted communication scenarios. The induced randomness method achieves secure and efficient key generation without complex hardware or high mobility. These protocols also apply in dynamic environments, provided the wireless channel remains unchanged during each random value exchange. Chen et al. [22] proposed a physical layer key generation scheme for WLAN MIMO systems based on feature fusion autoencoder (FFAEncoder) to address the issue of high key disagreement rate (KDR). This method extracts amplitude and phase features from CSI separately, fuses them through multiplication operations in a neural network and uses an autoencoder to extract standard features. Evaluations on multiple real-world scenario datasets show that the mean squared error (MSE) and mean absolute error (MAE) of the codewords between the transmitter and receiver are lower than those of current models, demonstrating better key generation performance. Chen et al. [23] proposed a Physical Layer Key Generation (PLKG) scheme based on Bidirectional Convergence Feature Learning (BCFL), which processes the obtained CSI data through deep learning to enable legitimate communication devices to acquire highly correlated channel characteristics during channel estimation. The BCFL scheme has advantages in execution time and Key Generation Rate (KGR), and it enhances the utilization efficiency of channel characteristics during the feature quantization stage through a multi-level quantization method. Wunder et al. [24] proposed the Full Duplex–Bisparse Blind Deconvolution (FD–BBD) scheme, which uses full duplex communication and bisparse blind deconvolution technology to achieve secure and efficient key generation in Wireless Personal Area Networks (WPANs). This method leverages the reciprocity of the wireless channel, rather than the channel’s entropy, to generate secret keys, making it suitable for various wireless environments, including those with high noise and signal fading. Through full duplex communication, legitimate parties can agree on a common secret key without channel probing. The current key distribution methods are limited mainly by their complex key management, computational overhead, and memory overhead, making them difficult to implement.

### 2.2. Introduction on Key Negotiation and Quantization

The application of key negotiation quantization technology in WSNs has attracted widespread attention. The primary goal is to optimize the key negotiation process under limited energy and bandwidth conditions, ensuring network security and improving communication efficiency. Due to the resource constraints of WSN nodes, traditional key negotiation protocols are not always applicable. Quantization technology plays a critical role in reducing communication overhead and energy consumption. In recent years, with the widespread application of WSNs, the use of quantization technology in key negotiation has further developed. Cao et al. [25] proposed an innovative key generation method that skips the traditional privacy amplification step and relies on multi-bit quantization and sample position coordination techniques. This method not only increases the key generation rate but also significantly enhances the security and consistency of the key through sample decorrelation. Hua [26] introduced the Generalized Channel Probing (GCP) and Generalized Preprocessing (GPP) technologies, which not only optimize channel capacity in non-reciprocal channel environments but also improve key generation efficiency in multi-antenna systems, breaking the dependency on channel reciprocity for key generation. Li et al. [27] addressed the issues of non-reciprocity and high correlation in OFDM channels by proposing a preprocessing mechanism based on Principal Component Analysis (PCA). Through the optimized preprocessing of channel measurement data, the performance of quantization and information reconciliation was significantly improved, ensuring a higher key generation rate and randomness. Wang et al. [28] introduced randomness into the quantization process by employing Hadamard matrices, significantly reducing the correlation of quantized bits and lowering the risk of malicious inference during public information exchange, achieving breakthroughs in key generation security. Li et al. [29] further refined channel preprocessing techniques by using PCA to eliminate the autocorrelation of channel samples, which not only improved the key generation rate and consistency but also ensured that the generated keys possessed high randomness and security, significantly optimizing subsequent information reconciliation and privacy amplification processes.

### 2.3. Introduction and Current State of Research on Negative Databases

A negative database is a data representation scheme inspired by artificial immune systems, which is distinct from traditional positive databases in that it stores the complement of information in the positive database. Given that negative databases are fully equivalent to an SAT (Boolean satisfiability) problem [30,31], and since SAT has been proven to be an NP-hard problem [32,33], retrieving the original data from a negative database is exceedingly tricky.

Existing algorithms for generating NDBs primarily include the clause distribution control algorithm [34], the 1-hidden algorithm [35], the 2-hidden algorithm [35], the q-hidden algorithm [33], the hybrid algorithm [36], the p-hidden algorithm [37], and the K-hidden algorithm [38].

Negative databases typically consist of binary strings. Suppose the positive database DB is composed of *n*-bit binary strings, U is the universe of *n*-bit binary strings, and U-DB is the complement of DB. The NDB stores a compressed form of the contents in U-DB. An example of a simple NDB is shown in Table 1, where * represents a wildcard that can denote either 0 or 1.

Existing NDB generation algorithms are primarily based on binary data. This paper proposes a real-valued NDB generation method for key synchronous generation, moving beyond the conventional binary NDB generation. In NDPCS-KS, generating communication keys involves one-way hash functions and a dual negotiation based on the NDB. Given the unidirectional nature, collision resistance of one-way hash functions, and the NP-hardness of reversing the NDB [32,33], essential information for communication key agreement is well protected. Even if an attacker obtains an NDB from a session (data transmission cycle), deducing the initial key seeds that generated the NDB is computationally infeasible, ensuring data security. Meanwhile, it is hard for attackers to generate legitimate communication keys due to the second negotiation through the NDB, since the attackers do not have the NDB and the negotiation key for second negotiation.

## 3. System Model

**Figure 1:**
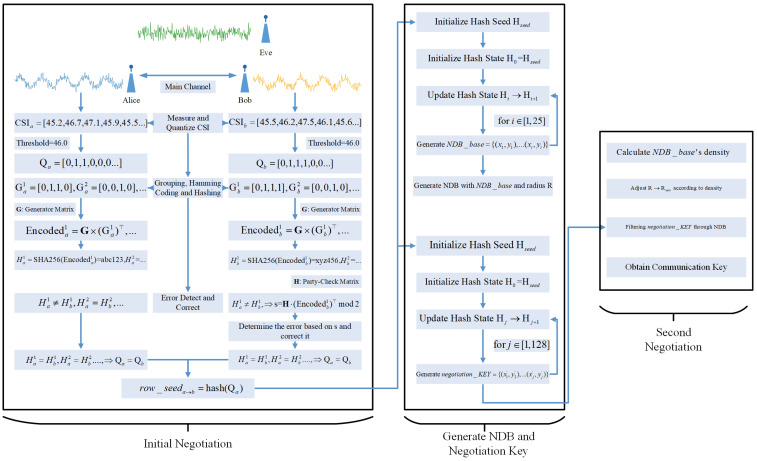
System model.

Figure 1 illustrates the overall framework of NDPCS-KS. First, the two communication nodes generate an initial key through the initial negotiation process. Specifically, both parties probe their CSI via the main channel. After quantization, the data are divided into groups, and the grouped quantized information is encoded using Hamming codes and then hashed. The hashed Hamming-encoded information is exchanged over the main channel, and both parties locally compare the results. If discrepancies are detected, indicating an error, error correction is performed by flipping the erroneous bits based on the parity check matrix calculated from the Hamming codes. Through this process, all groups undergo verification and error correction, ensuring that both parties ultimately generate the same initial key seed.

Next, using the initial key seed, an initial hash state is generated. This state is iteratively updated to generate the baseline point elements (base_point) of the NDB and the negotiation key (negotiation_KEY). The negotiation_KEY contains a series of negotiation values (negotiation_value). For each base_point, a circle with a fixed radius R is drawn, forming the NDB.

Finally, the communication key is generated through the second negotiation process. First, the density of the negotiation_value around each base_point is calculated, and based on this density, the radius R is dynamically increased or decreased to obtain a new radius $R_{new}$, making the final filtering more random. After dynamically adjusting the radius, each negotiation_value in the negotiation_KEY is used for filtering in the NDB. Specifically, if a negotiation_value falls within the circle defined by the radius around a base_point, the corresponding key position is set to 1. Conversely, if a negotiation_value does not fall within any circle, the corresponding key position is set to 0. This process results in a communication key with the same length as the negotiation_KEY.

Other more important definitions are shown in Table 2.

## 4. Key Technologies and Algorithms

### 4.1. Network Initialization

In the initialization phase of the sensor network, each node (sensor) sends pilot signals (heartbeat packets) to its directly connected neighboring nodes along the network topology. Then, these nodes broadcast response signals along the same topological paths, allowing each node in the network to obtain the initial CSI, specifically the RSSI (Received Signal Strength Indicator), of its directly connected neighboring nodes. RSSI is chosen as the CSI value due to its ease of measurement and robustness in dynamic environments. Although RSSI may have lower entropy compared to other CSI values such as phase information, it is stable and can effectively reflect signal strength variations caused by network topology changes. Moreover, due to the isolation characteristics of the physical channels, it is difficult for attackers to detect the RSSI values of the main channels through their eavesdropping channels.

Based on the RSSI value, the sensors can generate corresponding initial key seeds through initial negotiation with neighboring nodes. The initial negotiation is a process in which both parties negotiate initial key seeds using RSSI detected by each other. The two communicating parties typically synchronize the initial key seeds through the following four steps: (1) Measurement and Quantization of RSSI; (2) Grouping, Hamming Encoding and Hashing; (3) Error Detection and Correction; and (4) Generation of the Initial Key Seeds.

1. Measurement and Quantization of CSI Samples (RSSI): A pair of sensors (a,b) measures their CSI samples contained in the received pilot signals as follows:
  (1) $${\mathrm{CSI}}_{a}={\left\{{\mathrm{CSI}}_{a}\left[i\right]\right\}}_{i=1}^{N},\;{\mathrm{CSI}}_{b}={\left\{{\mathrm{CSI}}_{b}\left[i\right]+n\left[i\right]\right\}}_{i=1}^{N}$$
  where ${\mathrm{CSI}}_{a}\left[i\right]$ and ${\mathrm{CSI}}_{b}\left[i\right]$ represent the *i*th CSI sample measured by sensors *a* and *b*, respectively. n[i] represents noise, and *N* is the number of samples. Due to the reciprocity of the channel, sensors *a* and *b* can detect highly correlated CSI measurements, which exhibit similar fluctuation patterns. These patterns are primarily determined by the distance and interference factors in the physical channel connecting *a* and *b*. Suppose an attacker, Eve, is beyond the coherence distance from either *a* or *b*. She will observe entirely different fluctuation patterns [39,40], preventing her from eavesdropping on similar CSI and thus synchronizing the same initial key seed.
  The measured CSI samples are then coded into binary sequences as follows:
  (2) $$Q_{a}={\left\{q_{a}\left[i\right]\right\}}_{i=1}^{N}\;,\;Q_{b}={\left\{q_{b}\left[i\right]\right\}}_{i=1}^{N}$$
  where $q_{a}\left[i\right]$ and $q_{b}\left[i\right]$ represent the *i*th quantized CSI value of sensors *a* and sensor *b*, respectively. $q_{a}\left[i\right]=1$ if ${\mathrm{CSI}}_{a}\left[i\right]>\mathrm{threshold}$, otherwise $q_{a}\left[i\right]=0$.
2. Grouping, Hamming Encoding, and Hashing: The quantized CSI sequences are divided into multiple groups as follows:
  (3) $$G_{a}^{k}={\left\{q_{a}\left[i\right]\right\}}_{i=(k-1)\cdot m+1}^{k\cdot m}\;,\;G_{b}^{k}={\left\{q_{b}\left[i\right]\right\}}_{i=(k-1)\cdot m+1}^{k\cdot m}$$
  where $G_{a}^{k}$ and $G_{b}^{k}$ represent the *k*th group of quantized CSI values of sensor *a* and sensor *b*, respectively. *k* is the group index, and *m* is the number of samples per group. Each group of data is then encoded using the Hamming code (7,4) as follows:
  (4) $$\begin{array}{c} {\mathrm{Encoded}}_{a}^{k}=\mathrm{HammingEncode}\left(G_{a}^{k}\right)\\ {\mathrm{Encoded}}_{b}^{k}=\mathrm{HammingEncode}\left(G_{b}^{k}\right). \end{array}$$
  The Hamming code (7,4) is chosen for its single-bit error correction, making it suitable for mild noise in sensor networks. It provides high efficiency with low complexity, which is ideal for resource-constrained devices. In the network environment considered, where channel variation is minimal, Hamming ensures reliable communication without added overhead. In more complex environments, stronger error correction codes like Reed–Solomon or LDPC may be needed for higher noise and error levels. Then, the encoded groups are hashed as follows:
  (5) $$H_{a}^{k}=\mathrm{hash}\left({\mathrm{Encoded}}_{a}^{k}\right)\;\;H_{b}^{k}=\mathrm{hash}\left({\mathrm{Encoded}}_{b}^{k}\right)$$
  where $H_{a}^{k}$ and $H_{b}^{k}$ represent the hash values of the *k*th encoded group of sensors *a* and *b*, respectively.
3. Error Detection and Correction: When sensors *a* and *b* exchange hash values to detect inconsistencies between groups, if $H_{a}^{k}\neq H_{b}^{k}$, it indicates that group *k* contains errors. In such cases, Hamming decoding and correction are applied. Specifically, the syndrome $s_{i}$ is calculated using
  (6) $$s_{i}=H\cdot {\mathrm{Encoded}}_{b}^{k}{\left[i\right]}^{⊤}\;\mathrm{mod}\;2$$
  where H is the parity-check matrix of the Hamming code. The matrix H is used to determine the positions of errors in the encoded data by comparing it with the transmitted codeword. If the syndrome $s_{i}$ is non-zero, it indicates the presence of an error. The position of the error *j* is located, and the error is corrected by flipping the bit:
  (7) $${\mathrm{Encoded}}_{B}^{k}\left[j\right]\leftarrow {\mathrm{Encoded}}_{B}^{k}\left[j\right]⊕1.$$
  Equation (7) ensures that inconsistencies between the hash values are detected and corrected appropriately.
4. Generation of the Initial Key Seeds: After correcting all errors, both sensors concatenate the corrected CSI sequences and hash the result to generate shared initial key seeds. The concatenation of all corrected groups is expressed as
  (8) $$Q_{b}^{\mathrm{corrected}}=\overset{K}{\underset{k=1}{⋃}}G_{b}^{k}$$
  where $G_{b}^{k}$ represents the corrected sequence of group *k*, and *K* represents the total number of the groups. The shared initial key seed ${row\_seed}_{a\to b}$ is then generated by hashing the concatenated result:
  (9) $${row\_seed}_{a\to b}=\mathrm{hash}\left(Q_{b}^{\mathrm{corrected}}\right).$$

Through the above four steps, sensors *a* and *b* can synchronously generate the same initial key seeds, and it ensures that no confidential information is leaked to the attackers through eavesdropping on the wireless channels.

Then, each sensor will store all generated initial key seeds in a dictionary-type data structure called RS_Library. For example, for a sensor *a*, its RS_Library is as follows:

(10) $$RS\_Library=\begin{array}{c} \left[a\to b:{row\_seed}_{a\to b},\right.\\ \;\left.d\to a:{row\_seed}_{d\to a},\right.\\ \;\left.f\to a:{row\_seed}_{f\to a},\ldots \right]. \end{array}$$

Equation (10) indicates that sensor *b* is the parent of sensor *a*, and sensors *d* and *f* are the children of sensor *a* (that is, the child node is on the left side of → and the parent node is on the right side of →). This identification reflects the relationship between connected nodes. After the initial negotiation, each sensor establishes its local RS_Library, which is then used for subsequent key synchronization.

### 4.2. Negative Databases and Negotiation Key Generation

In order to build the same NDB for key synchronization in a pair of communication nodes (child,parent), they retrieve the local RS_Library for $row\_seed_{child\to parent}$. Then, the calculation of the two-dimensional baseline points of the NDB, $base\_point=(x_{i},y_{i})$, is conducted. The NDB consists of a set of filters, each comprising a base_point and a filtering radius. It should be noted that although the base_point calculation can be easily extended to high-dimensional spaces, the NDB baseline points are generated in a two-dimensional space to reduce the computational cost for WSNs.

#### 4.2.1. Negative Database Generation

Assume that there are two communication nodes, *a* and *b*, with *a* being the child node of *b*. The NDB_base is obtained through the following four steps.

1. Initialization and Generating the Initial Hash Seed: Initially, *a* and *b* retrieve ${row\_seed}_{a\to b}$ in their local RS_Library, respectively. Then, $H_{seed}$ is calculated based on ${row\_seed}_{a\to b}$:
  (11) $$H_{seed}=\mathrm{SHA}256\left({row\_seed}_{a\to b}\right).$$
2. Setting the Initial Hash State: Set the initial hash state $H_{0}$ to $H_{seed}$:
  (12) $$H_{0}=H_{seed}.$$
3. Generating $H_{i+1}$ as follows:
  (13) $$H_{i+1}=\mathrm{SHA}256\left(\mathrm{concat}\left(H_{i},H_{seed}\right)\right)$$
  in which the current hash state $H_{i}$ and $H_{seed}$ are employed to generate the next hash state $H_{i+1}$. Here, concat(*x*, *y*) concatenates two inputs, *x* and *y*, by appending the second input *y* to the first input *x*.
4. Generating the Coordinate Pairs $\left(x_{i},y_{i}\right)$ for NDB_base: the coordinates $x_{i}$ and $y_{i}$ are generated as follows:
  (14) $$\begin{array}{c} NDB\_base=\left\{\left(x_{i},y_{i}\right)|\right.\\ \;x_{i}=Round\left(\frac{int\left(hex\left(H_{i+1}\right)\left[:8\right]\right)}{16^{8}-1}\times 10,5\right),\\ \;y_{i}=Round\left(\frac{int\left(hex\left(H_{i+1}\right)\left[9:16\right]\right)}{16^{8}-1}\times 10,5\right),\\ \;i=1\ldots 25\}. \end{array}$$
  here, the Round(x,n) function rounds the number *x* to *n* decimal places. int(x) function converts a string *x* representing a hexadecimal number (base 16) into a decimal integer (base 10). hex(H) function generates the hexadecimal representation of the hash value *H*.

**Figure 2:**
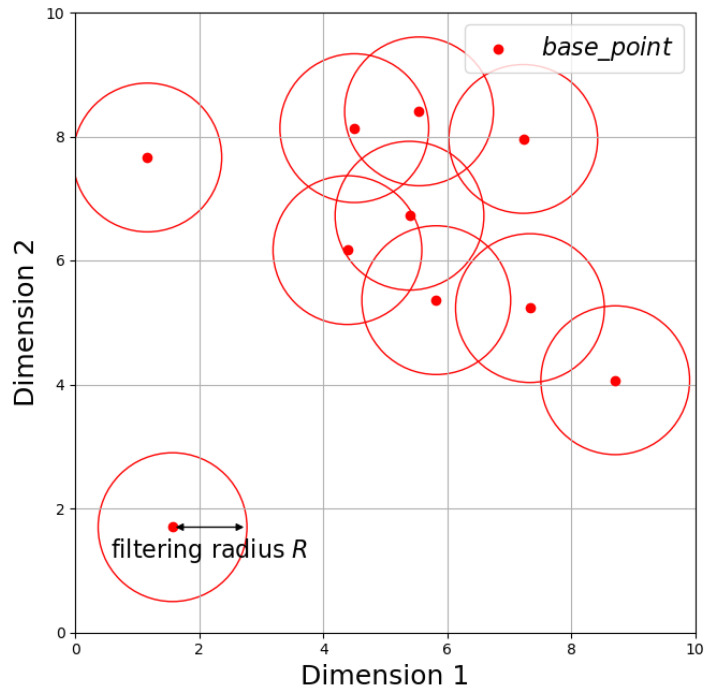
Schematic diagram of the NDB.

Through the aforementioned steps, NDB_base is generated. Then, with a constant radius *R*, circles are drawn centered at each base_point element in the NDB_base list, forming a two-dimensional filter library used as the NDB. Each base_point with a filter radius is referred to as a filter. Figure 2 is an example of an NDB, illustrating only ten filters for clarity.

Then, let us analyze the range of values for $x_{i}$ and $y_{i}$.

- For $int(hex\left(H_{i+1}\right)\left[:8\right])$
  1. “$hex(H_{i+1})$” returns a 64-character hexadecimal string.
  2. “[:8]” extracts the first eight characters, and these eight characters have a hexadecimal value range from “00000000” to “FFFFFFFF”.
  3. “int(…,16)” converts the hexadecimal string to a decimal integer with values ranging from 0 to $16^{8}-1$ (i.e., $2^{32}-1$).
- For $\frac{int(hex\left(H_{i+1}\right)\left[:8\right])}{16^{8}-1}$ and $\frac{int(hex\left(H_{i+1}\right)\left[9:16\right])}{16^{8}-1}$
  1. $hex\left(H_{i+1}\right)\left[:8\right]$ extracts the first eight characters for calculating $x_{i}$.
  2. $hex\left(H_{i+1}\right)\left[9:16\right]$ extracts the next eight characters for calculating $y_{i}$.
  3. Since SHA256 generates a 64-character hexadecimal hash value, and each character represents 4 bits, totaling 256 bits (32 bytes). Extracting any eight characters (32 bits) is sufficient to represent any value between 0 and $16^{8}-1$ (i.e., $2^{32}-1$), fully covering the required range.
  4. This ratio ranges from 0 to 1 because the numerator’s maximum value is $16^{8}-1$ (i.e., $2^{32}-1$) and the denominator is also $16^{8}-1$.
- Result after Multiplying by 10
  1. Multiplying the above ratio by 10 scales the range to 0 to 10.
- Final Rounding to 5 Decimal Places
  1. The “Round(…, 5)” function rounds the value to five decimal places. Therefore, the final values of $x_{i}$ and $y_{i}$ range from 0.00000 to 9.99999.

#### 4.2.2. Negotiation Key Generation

Next, the two-dimensional $negotiation\_value=(x_{j}^{\prime },y_{j}^{\prime })$ is generated in a manner similar to the base_point elements. For the *j*th instance, the current hash state $H_{j}$ is first updated, and the new hash value is computed as

(15) $$H_{j+1}=\mathrm{SHA}256\left(concat\left(H_{j},H_{seed}+byte\left[j\%256\right]\right)\right)$$

where byte[j%256] generates a byte with a value of jmod256. Subsequently, the negotiation_KEY is generated as follows:

(16) $$\begin{array}{c} negotiation\_KEY=\left\{\left(x_{j}^{\prime },y_{j}^{\prime }\right)|\right.\\ \;x_{j}^{\prime }=Round\left(\frac{int\left(hex\left(H_{j+1}\right)\left[:8\right]\right)}{2^{32}-1}\times 10,5\right),\\ \;y_{j}^{\prime }=Round\left(\frac{\mathrm{int}\left(hex\left(H_{j+1}\right)\left[9:16\right]\right)}{2^{32}-1}\times 10,5\right),\\ \;j=1\ldots 128\}. \end{array}$$

**Figure 3:**
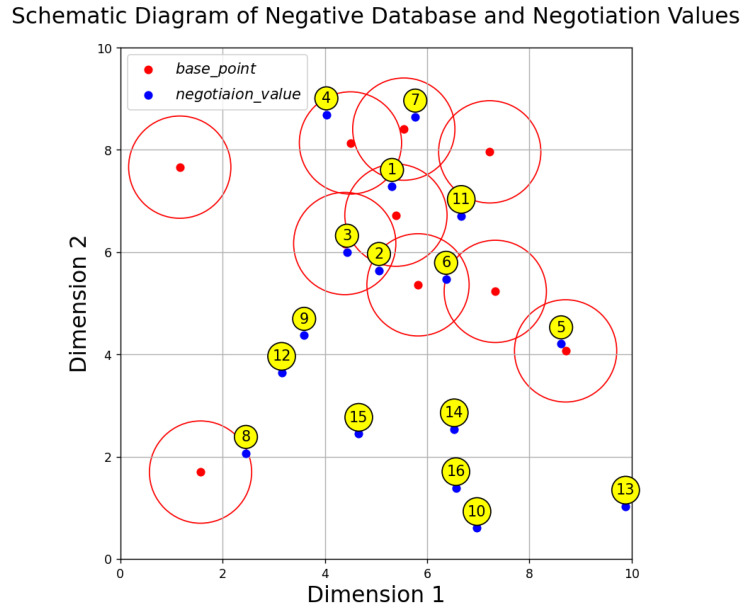
Schematic diagram showing the distribution of the generated NDB and negotiation key in a two-dimensional plane.

Figure 3 is a schematic diagram showing the distribution of the generated NDB and negotiation key in a two-dimensional plane. The numbers in the yellow circles represent the indices of the negotiation_value.

### 4.3. Second Negotiation for Communication Key Generation

After generating the NDB and negotiation key, a second negotiation is conducted for the negotiation key, using the NDB to filter the negotiation_KEY generated by Equation (16). Considering that the distribution of negotiation_value around each base_point varies, some base_point values may have an excessive concentration of negotiation_value, while others may have sparser distributions. This uneven distribution may lead to the communication keys being filtered with a fixed radius lacking sufficient randomness. Therefore, dynamic radius adjustment is introduced to fine-tune the filtering radius based on the density of negotiation_value around each base_point.

First, the density of negotiation_value around each base_point in the NDB is calculated. The filtering radius is dynamically adjusted based on the density to filter the negotiation_value. For each base_point, the number of negotiation_value within a given initial radius *R* is counted, and this number is divided by the area of the circular region $\left(\pi \times R^{2}\right)$ to obtain the density value. The radius *R* is an empirical value that needs to be adjusted according to actual conditions to ensure the randomness of the filtering process. The density calculation is as follows:

(17) $$\;\mathrm{density}\;=\frac{\;\mathrm{Number}\;\mathrm{of}\;\;negotiation\_value\;\mathrm{within}\;R}{\pi \times R^{2}}.$$

After obtaining the density of each base_point, the initial radius *R* is dynamically adjusted to a new filtering radius $R_{new}$. Specifically, suppose the density at a base_point exceeds a preset threshold $D_{high}$. In that case, the filtering radius at that base_point is reduced to minimize the impact of overly concentrated negotiation_value. Conversely, if the density is below a lower threshold $D_{low}$, the filtering radius is increased to capture more potentially relevant negotiation_value. By dynamically adjusting the filtering radius based on the density of negotiation_value around the base_point, the radius is decreased for high-density points and increased for low-density points, making the filtering process more random. Let $D_{high}$ be the high threshold, $D_{low}$ be the low threshold, and Δ be the adjustment value; then, the calculation of $R_{new}$ is as follows:

(18) $$R_{new}=\left\{\begin{array}{c} R+\Delta ,\;\mathrm{if}\;\mathrm{density}>D_{high}\\ R-\Delta ,\;\mathrm{if}\;\mathrm{density}<D_{low}\\ R,\;\mathrm{otherwise}. \end{array}\right.$$

Note that the initial filter radius *R* and parameters such as the adjustment value Δ and thresholds $D_{high}$ and $D_{low}$ for dynamically adjusting the radius are pre-shared among all nodes in the sensor network. After dynamically adjusting the filtering radius, the negotiation_value in the negotiation_KEY are filtered to generate the communication key. An empty key sequence filtered_key of the same length as the negotiation_KEY is created. For each $negotiation\_value=(x_{j}^{\prime },y_{j}^{\prime })$ (where j=1,2,…,128), if its distance to the nearest base_point is less than the corresponding filtering radius, it is covered (or filtered) by the NDB, and the corresponding position *j* in the key sequence is set to 1, filtered_key[j]=1; otherwise, it is set to 0, filtered_key[j]=0. Let $\left(x_{j}^{\prime },y_{j}^{\prime }\right)$ be the coordinates of the negotiation_value in the negotiation_KEY (where j=1,2,…,128), $\left(x_{B},y_{B}\right)$ be the coordinates of the nearest base_point, and $d=\sqrt{{\left(x_{j}^{\prime }-x_{B}\right)}^{2}+{\left(y_{j}^{\prime }-y_{B}\right)}^{2}}$ be the distance between the current $negotiation\_value=(x_{j}^{\prime },y_{j}^{\prime })$ and the nearest base_point$\left(x_{B},y_{B}\right)$. The filtering process is then shown below:

(19) $$filtered\_key\left[j\right]=\left\{\begin{array}{c} 1,\;\mathrm{if}\;d<R_{new}\\ 0,\;\mathrm{otherwise}\;. \end{array}\right.$$

**Figure 4:**
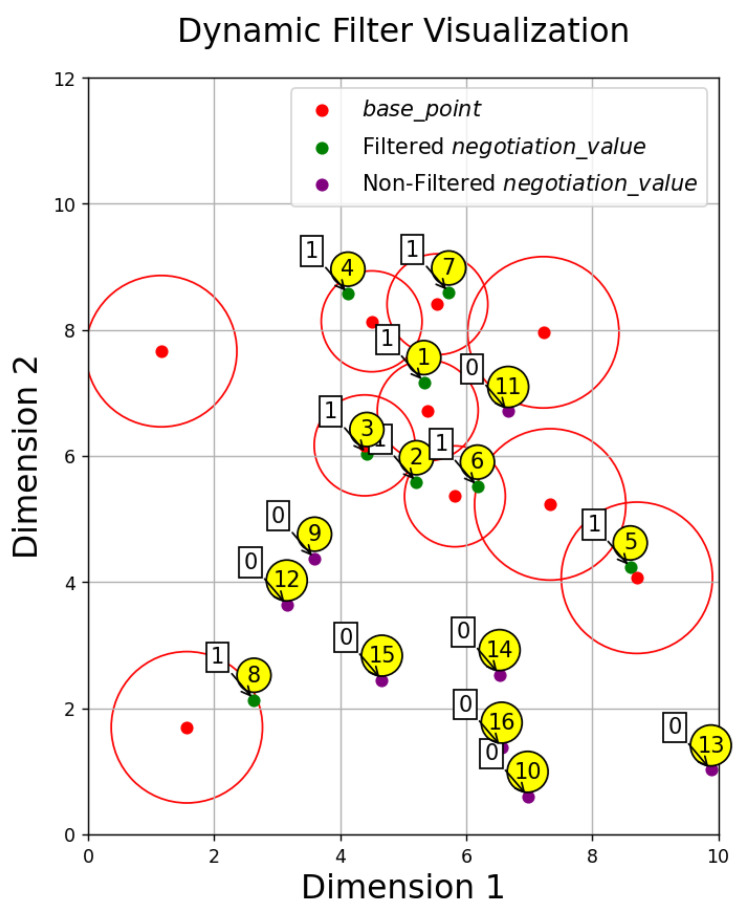
Schematic diagram of communication key generated by dual negotiation of the NDB.

Thus, by filtering the negotiation_KEY through a second negotiation using the NDB, a 128-bit key sequence filtered_key is obtained. This communication key sequence can be directly used for encryption and decryption with the length of the communication key being dynamically adjustable. The schematic diagram of the second negotiation process for generating the communication key is shown in Figure 4. In the figure, purple dots represent unfiltered negotiation_value, red dots represent base_point, green dots represent filtered negotiation_value, and red circles represent the dynamic radii corresponding to the base_point. The radii vary for different base_point due to dynamic radius adjustment based on the density of the negotiation_value near each base_point. The negotiation_value covered by the red circles are filtered. The numbers in yellow circles indicate the identification numbers of the negotiation_value, while the numbers in the squares represent the assignments in the filtered key sequence after filtering. For example, the communication key sequence filtered out in Figure 4 is 1111111100000000. Note that this example is for illustrative purposes, and the actual communication key generation is random.

The following Algorithm 1 describes the steps of the key generation method based on the NDB.

Algorithm 1 begins by taking $row\_seed_{a\to b}$ as input and computing its SHA256 hash value $H_{seed}$, which is then set as the initial hash state $H_{0}$ (Lines 1 and 3). An empty set NDB_base is initialized to store the base_point. The algorithm iterates from i=1 to i=25, updating the hash state from $H_{i}$ to $H_{i+1}$ using Equation (13), and calculating and storing the base_point in NDB_base using Equation (14) (Lines 4–9). Next, an empty set negotiation_KEY is initialized to store the negotiation_value. The algorithm iterates from j=0 to j=128, updating the hash state from $H_{j}$ to $H_{j+1}$ using Equation (15), and calculating and storing the negotiation_value in negotiation_KEY using Equation (16) (Lines 10–14). For each base_point in NDB_base, the algorithm calculates its density using Equation (17) and dynamically adjusts its radius using Equation (18) (Lines 15–18). An empty set filtered_key is then initialized to store the communication key. For each $negotiation\_value=(x_{j}^{\prime },y_{j}^{\prime })$ in negotiation_KEY, the algorithm filters to obtain filtered_key[j] using Equation (19). Finally, filtered_key is converted to a binary string $K_{a\to b}$, which is returned as the communication key.
**Algorithm** **1** Key Generation Method based on Negative Database1: **Input**: $row\_seed_{a\to b}$2: **Output**: Returns a byte array representing the communication key, $K_{a\to b}$ 3: $H_{seed}=\mathrm{SHA}256\left(row\_seed_{a\to b}\right)$4: $H_{0}=H_{seed}$5: NDB_base←∅6: **for** i←1 **to** 25 **do**7:    update the hash state $H_{i}$ to $H_{i+1}$ using Equation (13).8:    *NDB_base[i]* = $\left(x_{i},y_{i}\right)$, calculated using Equation (14).9: **end for**10: negotiation_KEY←∅11: **for** j←0 **to** 128 **do**12:    update the hash state $H_{j}$ to $H_{j+1}$ using Equation (15)13:    *negotiation_KEY[i]* = $\left(x_{j}^{\prime },y_{j}^{\prime }\right)$, calculated using Equation (16).14: **end for**15: **for** each *base_point* in *NDB_base* **do**16:    calculate the density of *base_point* using Equation (17).17:    dynamically adjust the radius of *base_point* using Equation (18).18: **end for**19: filtered_key←∅20: **for** each *negotiation_value* = $\left(x_{j},y_{j}\right)$ in *negotiation_KEY* **do**21:    filter $\left(x_{j},y_{j}\right)$ to obtain *filtered_key[j]* using Equation (19).22: **end for**23: $K_{a\to b}\leftarrow$ convert filtered_key to a binary string24: **return** $K_{a\to b}$

### 4.4. Identity Verification Mechanism Based on SHA256

Considering that an attacker may send forged information to a node in a sensor network or tamper with legitimate information from a node in a sensor network and send it to other nodes again, an identity verification mechanism based on SHA256 is designed to complete the identity verification of the encryption party.

Specifically, when sensor *a* is about to send an encrypted message to sensor *b*, sensor *a* needs to calculate an identity verification value $v_{a\to b}$ as follows:

(20) $$v_{a\to b}=\mathrm{SHA}256\left(\mathrm{encrypted}\;\mathrm{message}+row\_seed_{a\to b}\right).$$

In Equation (20), $row\_seed_{a\to b}$ is the initial key seed, and the encrypted message is the encrypted observation value. The hash verification value $v_{a\to b}$ is appended to the encrypted observation value and sent to sensor *b*. Sensor *b* extracts $v_{a\to b}$ and retrieves $row\_seed_{a\to b}$ from its RS_Library. Sensor *b* uses $row\_seed_{a\to b}$ and the encrypted message in Equation (20) to calculate $v_{a\to b}^{\prime }$. If $v_{a\to b}=v_{a\to b}^{\prime }$, $row\_seed_{a\to b}$ is used to generate the communication key $K_{a\to b}$ for decryption. Algorithm 2 details the identity verification mechanism based on SHA256.
**Algorithm** **2** Identity Verification Mechanism Based on SHA2561: **Input:** $row\_seed_{a\to b}$ and encrypted message2: **Output:** communication key $K_{a\to b}$ or false 3: $v_{a\to b}$ calculated using Equation (20)4: **for** each initial key seed in RS_Library of parent node *b* **do**5:    $v_{a\to b}^{\prime }$ calculated using Equation (20)6:    **if** $v_{a\to b}$ = $v_{a\to b}^{\prime }$ **then**7:        calculate $K_{a\to b}$ using Algorithm 18:        **return** $K_{a\to b}$9:    **else**10:        **return** false11:    **end if**12: **end for**

Algorithm 2 takes $row\_seed_{a\to b}$ and the encrypted message as inputs, and it calculates $v_{a\to b}$ using Equation (20) (Lines 1 and 3). The parent node *b* then search initial key seeds in its local RS_Library to calculate the identity verification value $v_{a\to b}^{\prime }$ using Equation (20) and compares it with $v_{a\to b}$ (Lines 4–5). If a $row\_seed_{a\to b}$ satisfies $v_{a\to b}=v_{a\to b}^{\prime }$, it is used as input for Algorithm 1 to calculate the communication key $K_{a\to b}$ for decryption (Lines 6–8). If no such $row\_seed_{a\to b}$ exists, it indicates that the encrypted information from child node *a* is either forged or tampered with (Lines 9–10). Since the attacker does not know $row\_seed_{a\to b}$, according to Equation (20), the attacker either tampered with the encrypted information or forged the $row\_seed_{a\to b}$.

### 4.5. Lightweight Anti-Replay Mechanism Based on Timestamps and Rolling Hash Tokens

Since WSNs broadcast data packets, encrypting observation data alone makes them susceptible to replay attacks. In critical applications, such as temperature monitoring in chemical plants, such attacks can have severe consequences. This section introduces a lightweight scheme based on timestamps and rolling hash tokens to achieve anti-replay with lower resource consumption.

Specifically, each node maintains a library of rolling hash tokens called RHT_Library, recording the most recent *n* legitimate tokens. When sensor *a* sends an encrypted message to sensor *b*, sensor *a* attaches a timestamp Time_Stamp that includes the generation time $T_{gen}$ and the token expiration time $T_{exp}$, which is denoted as follows:

(21) $$Time\_Stamp=T_{gen}\left|\right|T_{exp}$$

here, the token expiration time T_exp is the generation time T_gen plus a certain time. The Time_Stamp is encrypted with the communication key $K_{a\to b}$ as the encrypted message. Then, the encrypted timestamp $E(K_{a\to b},Time\_Stamp)$ is used as input for SHA256, which outputs a token TK as follows:

(22) $$TK=\mathrm{SHA}256(E\left(K_{a\to b},Time\_Stamp\right)).$$

The child node *a* sends the message group [encrypted message, $E(K_{a\to b},Time\_Stamp)$, TK, $v_{a\to b}$] to the parent node *b*. In the proposed anti-replay mechanism, replay attacks are detected under two circumstances. The first occurs when the $T_{exp}$ has already passed the current system time of the receiving node. In this case, it is considered a replay attack, as it is highly likely that the attacker is replaying a previously intercepted message at a different time. The second occurs when the $T_{exp}$ has not yet passed the current system time of the receiving node, but the TK appears in the RHT_Library maintained by the receiving node. This is also considered a replay attack, because after intercepting the message, the attacker attempts to quickly replay it to pass the expiration time check. However, since the RHT_Library stores the most recent valid tokens, the attacker’s attempt to replay the message quickly is likely to collide with the tokens in the receiving node’s RHT_Library, as the valid tokens have not yet been updated during this period.

Algorithm 3 describes the process of a lightweight anti-replay mechanism based on timestamps and rolling hash tokens.
**Algorithm** **3** Lightweight Anti-Replay Mechanism Based on Timestamps and Rolling Hash Tokens1: **Input:** TK, $E(K_{a\to b},Time\_Stamp)$, $v_{a\to b}$2: **Output:** replay attack detected or not 3: using $v_{a\to b}$ as input to Algorithm 2 to generate $K_{a\to b}$4: Time_Stamp ← $D(K_{a\to b},Time\_Stamp)$ using $K_{a\to b}$5: $T_{exp}$ ← Time_Stamp6: **if** $T_{exp}>$ current system time **then**7:    **return** replay attack detected8: **else**9:    **return true**10: **end if**11: **for** each token in RHT_Library of sensor *b* **do**12:    **if** token = TK **then**13:        **return** replay attack detected14:        **break**15:    **else**16:        push TK onto RHT_Library17:        **if** RHT_Library size exceeds limit *n* **then**18:           pop the oldest token from RHT_Library19:        **end if**20:    **end if**21:**end for**

In Algorithm 3, sensor *b* first extracts $v_{a\to b}$ from the message group [encrypted message, $E(K_{a\to b},Time\_Stamp)$, TK, $v_{a\to b}$]. This extracted $v_{a\to b}$ is used as input for Algorithm 2 to perform identity verification. If the identity is successfully verified, the communication key $K_{a\to b}$ is generated. Next, $K_{a\to b}$ is used to decrypt $E(K_{a\to b},Time\_Stamp)$ to obtain the Time_Stamp. The expiration time $T_{exp}$ is extracted from the Time_Stamp and compared with the current system time of sensor *b*. If $T_{exp}$ has already passed the current system time, the token is considered expired (Lines 3–10). If the token has not expired, the next validation step is performed. The TK is extracted from the message group and compared with each token in the RHT_Library maintained by sensor *b*. If a matching token TK is found in the RHT_Library, it indicates a replay attack (Lines 11–14). Conversely, if no matching token is found, the message is considered legitimate, and the communication token TK is stored in the RHT_Library. If the RHT_Library is full, the oldest token is removed to make space for the new token (Lines 15–21). By adjusting the size of the RHT_Library and the validity period of the tokens, a lightweight and efficient anti-replay mechanism can be implemented.

### 4.6. Data Transmission Process Between Nodes

**Figure 5:**
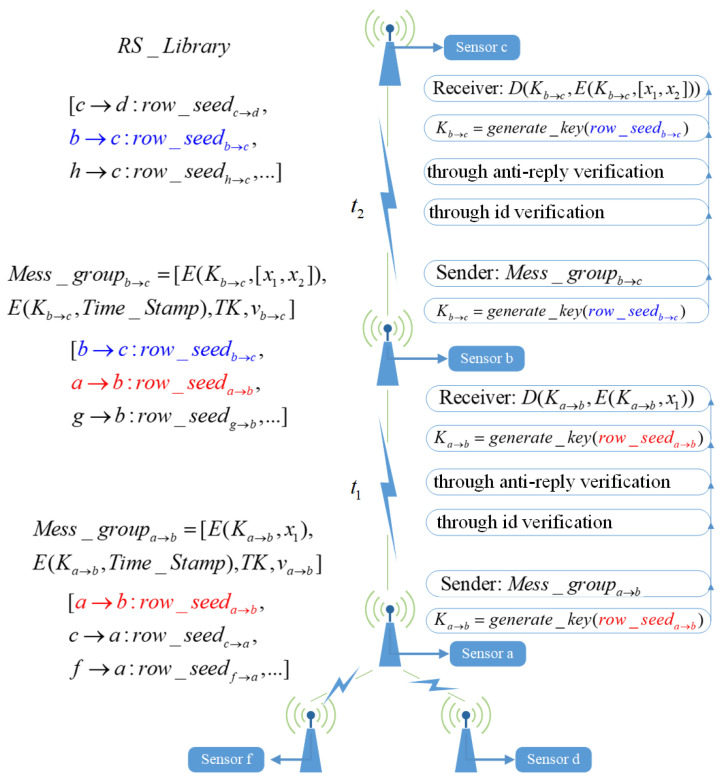
Data transmission flow.

Figure 5 illustrates an example sensor network topology and the steps of data transmission between sensors. In each session, each sensor transmits encrypted data to its parent node. Assume $\left[t_{1},t_{2},t_{3},\ldots ,t_{n}\right]$ represents the start times of each session. At time $t_{1}$, sensor *a* first derives the initial key seed ${row\_seed}_{a\to b}$ from the CSI value between sensor *a* and its parent node *b* during the initial negotiation. Then, the second negotiation is used to obtain the communication key $K_{a\to b}$. After generating $K_{a\to b}$, sensor *a* uses $K_{a\to b}$ to encrypt its observation $x_{1}$, resulting in the encrypted observation $E(K_{a\to b},x_{1})$. Subsequently, the identity verification value $v_{a\to b}$ is obtained using Equation (20), and the Time_Stamp is generated using Equation (21). The Time_Stamp is then encrypted, resulting in $E(K_{a\to b},Time\_Stamp)$. Next, the token TK is obtained using Equation (22). Finally, the above information is assembled into a message group $Mess\_group_{a\to b}=[E\left(K_{a\to b},x_{1}\right),E\left(K_{a\to b},Time\_Stamp\right),TK,v_{a\to b}$] and sent to sensor *b*.

Upon receiving the message group $Mess\_group_{a\to b}$ from sensor *a*, parent node *b* first extracts the identity verification value $v_{a\to b}$, the encrypted timestamp $E(K_{a\to b},Time\_Stamp)$, and the token TK. Then, sensor *b* executes identity verification using Algorithm 2 with the extracted $v_{a\to b}$ and then applies the lightweight anti-replay mechanism using Algorithm 3 with the extracted $E(K_{a\to b},Time\_Stamp)$ and TK. After successful verification and anti-replay checks, sensor *b* synchronizes the same communication key $K_{a\to b}$ through the second negotiation using ${row\_seed}_{a\to b}$ based on the CSI value at time $t_{1}$. Finally, sensor *b* uses the synchronized key $K_{a\to b}$ to decrypt the observation data by performing $D(K_{a\to b},E\left(K_{a\to b},x_{1}\right))$, accessing the observation data $x_{1}$ from child node *a* at time $t_{1}$.

Subsequently, at time $t_{2}$, parent node *b* integrates its observation data $x_{2}$ with the data from child node *a* into a data series $\left[x_{1},x_{2}\right]$. Node *b* then uses ${row\_seed}_{b\to c}$ to generate the communication key $K_{b\to c}$ for encryption. The encrypted data series $E(K_{b\to c},\left[x_{1},x_{2}\right])$, along with the identity verification value $v_{b\to c}$, the encrypted timestamp $E(K_{b\to c},Time\_Stamp)$, and the token TK, are assembled into a message group $Mess\_group_{b\to c}$. $Mess\_group_{b\to c}$ is then sent to the grandparent node *c*.

Upon receiving $Mess\_group_{b\to c}$, node *c* performs identity verification using Algorithm 2 and applies the lightweight anti-replay mechanism using Algorithm 3. After successful verification and anti-replay checks, node *c* synchronizes the communication key $K_{b\to c}$ using ${row\_seed}_{b\to c}$ at time $t_{2}$. Finally, node *c* decrypts the data series with $D(K_{b\to c},E\left(K_{b\to c},\left[x_{1},x_{2}\right]\right))$, accessing the observation data $\left[x_{1},x_{2}\right]$.

Repeat the above steps until all child nodes have safely transmitted their observation values to the final root node. After each key synchronization and successful encryption-decryption communication, both communicating parties will update their locally maintained RS_Library using SHA256 to update the initial key seed used in the current session. Specifically, considering sensors *a* and *b* as examples, sensor *a* uses $row\_seed_{a\to b}\left(t_{1}\right)$ at time $t_{1}$ to generate the communication key, encrypts the message, and transmits it to sensor *b*. Upon receiving the encrypted message, sensor *b* uses $row\_seed_{a\to b}\left(t_{1}\right)$ to generate the same communication key and successfully decrypts the message. Subsequently, both sensors *a* and *b* will update $row\_seed_{a\to b}\left(t_{1}\right)$ in their locally maintained RS_Library as follows:

(23) $$row\_seed_{a\to b}\left(t_{2}\right)=\mathrm{SHA}256\left(row\_seed_{a\to b}\left(t_{1}\right)\right)$$

where $row\_seed_{a\to b}\left(t_{2}\right)$ is the key seed that sensors *a* and *b* will use in the next session start time $t_{2}$. This ensures that each key seed is different in different sessions, generating a unique key for different sessions, achieving a “one-time pad”, and also ensuring that the keys generated by both communicating parties are synchronized.

It is important to note that the method proposed in this paper is suited for relatively static wireless sensor network environments and does not account for significant node mobility. In cases where nodes disconnect, since each node has already acquired and stored the CSI with its directly connected nodes during the initialization phase, the disconnection of certain leaf nodes will not affect the communication of the entire network. The security of communication relies on the CSI of the directly connected nodes rather than the global network topology. This ensures that even if some nodes leave, the remaining nodes can still communicate normally. However, if there are significant changes in the network topology, such as large-scale node disconnections or mobility that leads to substantial structural changes, the network will need to be re-initialized. This will involve re-acquiring the CSI and re-synchronizing the keys to ensure the security and reliability of communication.

## 5. Security Analysis

### 5.1. Key Space Size

The dual negotiation processes generate the communication key, including the initial negotiation using Algorithm 1 and the second negotiation through the filtering process by an NDB. For example, suppose the length of the final negotiation_KEY is 128 with each bit of the key having a 50% probability of being 0 or 1. In that case, the key space size is $2^{128}$, which is a considerable number sufficient to withstand brute force attack with current computing resources as it is a “one-time pad” and changes in different sessions. According to the randomness analysis in Section 6.4 it is evident that the distribution probability of each bit of the key approaches 50% for both 0 and 1. Therefore, the key space size of NDPCS-KS approaches $2^{128}$, which remains computationally infeasible for brute force attacks.

### 5.2. Key Security

In WSNs, attackers cannot obtain the CSI between the communicating parties due to the isolation of the physical channels. They thus cannot calculate the initial key seeds. It is computationally infeasible for attackers to derive a legitimate communication key without the initial key seeds, as demonstrated below:

- Let *S* be the initial key seed and let Hash() denote a hash function (specifically SHA256).
- Let $B=\left(x_{1},y_{1}\right),\left(x_{2},y_{2}\right),\cdots ,\left(x_{n},y_{n}\right)$ be a set of *n* baseline points generated based on *S*.
- Let $P=\left(x_{1}^{\prime },y_{1}^{\prime }\right),\left(x_{2}^{\prime },y_{2}^{\prime }\right),\cdots ,\left(x_{m}^{\prime },y_{m}^{\prime }\right)$ be a set of *m* negotiation value points generated.
- Assume the initial filtering radius is *R*, the adjustment value is Δ, the high density threshold is $D_{high}$, and the low density value is $D_{low}$. All these parameters are pre-shared by all nodes in the sensor network.

The attacker aims to deduce *P* filtered by *B* to derive the communication key without knowing *S*. For the attacker, without *S*, it is difficult to calculate *B* or *P* within an acceptable time frame (assuming a typical WSN session cycle is 15 min, after which a new session and key updating process begins) because the unidirectional nature of the hash function Hash() makes it computationally infeasible to deduce the input from its output. Since the attacker cannot effectively find two different inputs that lead to the same output, they cannot find a different seed $S^{\prime }$ to generate the same sets $P^{\prime }$ and $B^{\prime }$. It is computationally infeasible even if the attacker attempts to brute-force guess the valid *B* and *P*. For instance, $\left|B\right|=25$ and $\left|P\right|=128$ consist of two-dimensional coordinates within the range [0,10] × [0,10]. The coordinates for *B* and *P* are rounded to five decimal places, each with $10^{5}\times 10=10^{6}$ possible values (from 0.00000 to 9.99999). For the base_point set *B*, each base_point has $10^{6}\times 10^{6}=10^{12}$ possible positions, and since *B* contains 25 such points, the potential space size of *B* is ${\left(10^{12}\right)}^{25}$. For the negotiation_value set *P*, each member also has $10^{12}$ possible positions, and since *P* includes 128 such points, the potential space size of *P* is ${\left(10^{12}\right)}^{128}$. The attacker would need to simultaneously guess *B* and *P*, leading to a total potential space size of ${\left(10^{12}\right)}^{25}\times {\left(10^{12}\right)}^{128}=10^{1836}$, which is clearly computationally infeasible.

Furthermore, due to the second negotiation through the NDB, since the attacker does not know the initial filtering radius *R*, the adjustment value Δ, or the density thresholds $D_{high}$ and $D_{low}$, it is tough to filter out a legitimate communication key.

In conclusion, an attacker cannot calculate and obtain the communication key without the initial key seed *S* within an acceptable time frame.

## 6. Experiments

**Figure 6:**
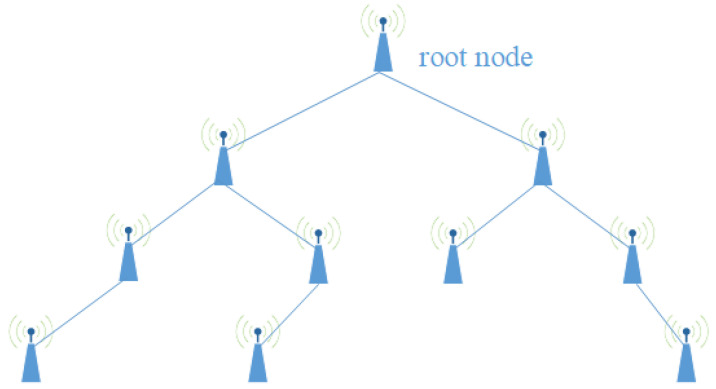
Schematic diagram of sensor topology.

**Figure 7:**
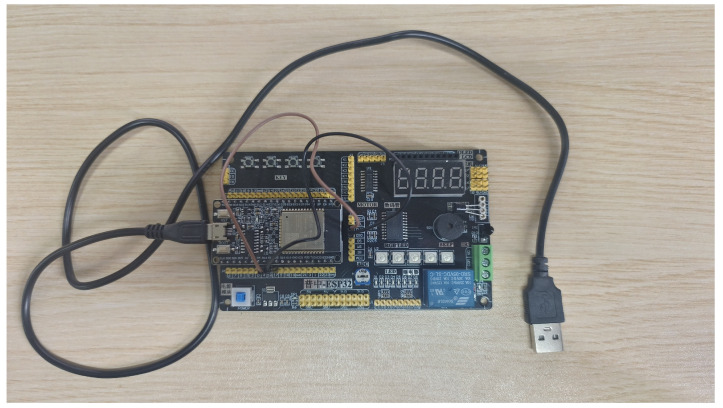
Schematic diagram of ESP32 development board.

The experiment arranged 10 ESP32 development boards with sensors into a tree topology, as shown in Figure 6. Every 30 s, the sensors generate a simulated observation value of 0 or 1. Following the procedure described in Section 4.6, after generating the key, the observation values are encrypted using SIMON [41], forming the corresponding message group, and sent to the parent node via the ESP-NOW protocol. Regarding the parameters of NDPCS-KS, the filter radius *R* is set to 1.5, the dynamic adjustment value Δ=0.25, and there are 25 base_point elements and 128 negotiation_value. The ESP32 development board used in the experiment is shown in Figure 7.

The experiments include the following tasks:

1. Simulated replay attacks and calculated detection accuracy.
2. Simulated attacks involving tampering and forging transmission packets and calculated detection accuracy.
3. Analyzed the performance of NDPCS-KS, recorded the average key generation time, and compared its performance with the key generation method described in references [18,19,20].
4. The randomness of NDPCS-KS was validated through probabilistic statistics, Monte Carlo simulations, and entropy statistics experiments. Additionally, the NIST Statistical Test Suite [42] was used to test the randomness of the keys generated by NDPCS-KS.

### 6.1. Replay Attack Detection Accuracy

Select any two sensors, *a* and *b*, and let sensor *a* send 1000 data messages to sensor *b* with the attacker intercepting the sent data with a probability of 0.2 and replaying it after a random delay. The expiration time $T_{exp}$ in each data timestamp is set to the current system time +60s at the time of timestamp generation $T_{gen}$. Each sensor maintains a rolling hash token library RHT_Library storing the most recent ten legitimate tokens. When a replay attack is detected, a counter is incremented by 1, and the accuracy of replay attack detection can be calculated as follows:

(24) $$\mathrm{detection}\_\mathrm{rate}=\frac{\mathrm{replay}\_\mathrm{attack}\_\mathrm{detected}}{1000\times 0.2}.$$

**Figure 8:**
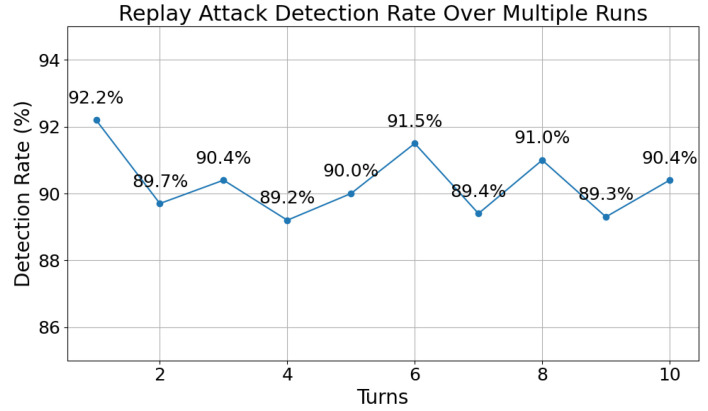
Replay attack detection accuracy.

The experiment was repeated ten times, resulting in the outcomes shown in Figure 8.

The results show that the detection accuracy is around 90%, with the highest reaching 92.2%, indicating effective performance.

More accurate anti-replay can be achieved by setting the size of the RHT_Library and the $T_{exp}$ appropriately. For instance, in a specific WSN environment, let the size of the RHT_Library maintained by each sensor be *n*, and let the communication frequency between sensors be λ (representing the average number of token requests per unit time). To maximize the detection rate of replay attacks, the condition must be satisfied as follows:

(25) $$T_{exp}\leq \frac{n}{\lambda }.$$

Equation (25) required that the token’s lifetime $T_{exp}$ should be less than or equal to the number of tokens expected to be received by the system during its lifetime $\frac{n}{\lambda }$. This way, once a token expires, it is no longer in the RHT_Library (as new tokens have replaced it), and any unexpired replay tokens will also be recognized because they are already in the RHT_Library. Effective anti-replay can be achieved by customizing the expiration time $T_{exp}$ and the size of the RHT_Library *n* according to the communication frequency λ of different WSNs.

### 6.2. Tampering and Forging of Transmission Packets Detection Accuracy

**Figure 9:**
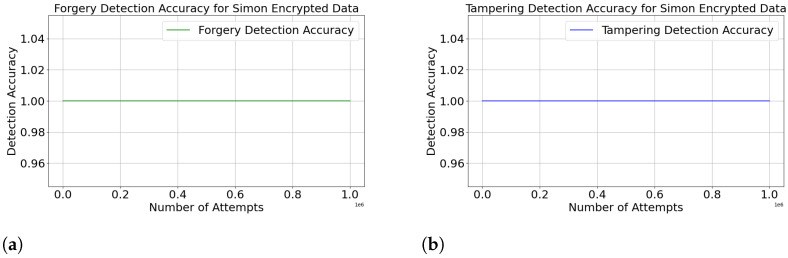
(**a**) Forgery detection accuracy. (**b**) Tamper detection accuracy.

The experiment uses a lightweight SIMON algorithm for encryption. After intercepting encrypted data packets sent by sensor *a* to sensor *b*, the attacker randomly tampers with part of the packet content, forming a tampered data packet. Moreover, the attacker uses randomly generated CSI to obtain his own $row\_seed_{attacker\to b}$ and encrypts the forged observation values before sending them to sensor *b*. By counting the number of times sensor *b* reports the identity verification mechanism based on SHA256 returns falsely, it is possible to determine whether the data packets have been tampered with or forged and calculate the corresponding detection accuracy. Figure 9 shows the detection accuracy for forged and tampered data.

Forgery and tampering were conducted 1,000,000 times each, and as shown in Figure 9, the detection accuracy for both forgery and tampering is 100%. This is because the SHA256 hash function is unidirectional and highly resistant to collisions. This means that even if an attacker can access the hash verification value $v_{a\to b}$, he cannot deduce the initial key seeds or encrypted message nor modify the encrypted message without detection. Furthermore, the verification value $v_{a\to b}$ is generated based on the ciphertext and a secret seed; the correct $v_{a\to b}$ can only be generated when both are unaltered. Any modification to the ciphertext or seed will result in a verification failure.

### 6.3. Performance Analysis

**Figure 10:**
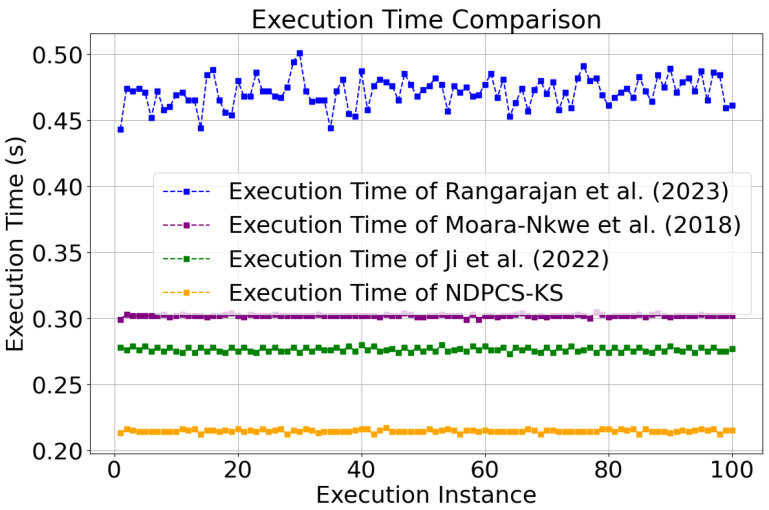
Comparison of execution time. The execution time of NDPCS-KS is compared with that of Rangarajan et al. (2023) [18], Moara-Nkwe et al. (2018) [19], and Ji et al. (2022) [20].

NDPCS-KS (generating a 128-bit key) and the method from references [18,19,20] are carried out 100 times on sensors. Record the time cost of each execution, calculate the average execution time of 100 key generations, and plot, as shown in Figure 10. As can be seen from Figure 10, the average execution time of NDPCS-KS is 0.21449 s, which is faster than 0.47139 s in reference [18], 0.30205 s in reference [19], and 0.27638 s in reference [20].

**Figure 11:**
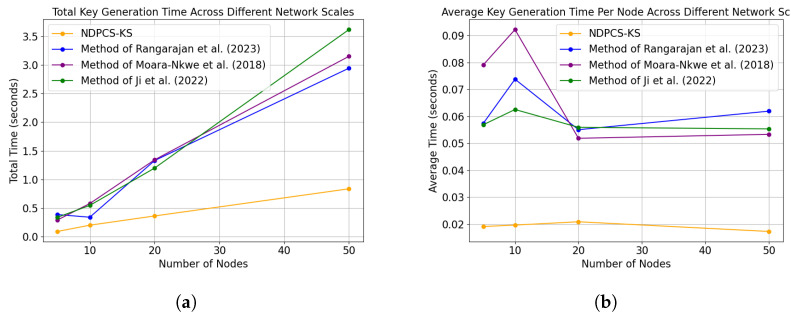
(**a**) Total key generation time across different network scales. The execution time of NDPCS-KS is compared with the methods of Rangarajan et al. (2023) [18], Moara-Nkwe et al. (2018) [19], and Ji et al. (2022) [20]. (**b**) Average key generation time per node across different network scales. The comparison includes NDPCS-KS and the methods from Rangarajan et al. (2023) [18], Moara-Nkwe et al. (2018) [19], and Ji et al. (2022) [20].

Due to experimental limitations, a simulation-based approach on a computer was adopted to compare the performance overhead of various methods under different network scales. Figure 11 presents the total time overhead for key generation and the average time overhead per node for key generation under different network scales for the methods referenced in [18,19,20] as well as NDPCS-KS.

As shown in Figure 11, NDPCS-KS demonstrates better performance and computational complexity across different network scales. Specifically, the average key generation time per node is consistently lower compared to the methods in references [18,19,20], indicating that NDPCS-KS reduces the computational burden on individual nodes. Furthermore, the total key generation time across the network scales also shows a clear advantage, suggesting that NDPCS-KS scales more efficiently as the network size increases. This reduction in both individual and total key generation times reflects a decrease in computational complexity, which in turn implies lower energy consumption, making NDPCS-KS more suitable for resource-constrained environments like sensor networks.

### 6.4. Randomness Analysis

#### 6.4.1. Probabilistic Statistics

A secure key generation method is expected to exhibit randomness and unpredictability, meaning the probability of each bit of the generated key being 0 or 1 should approach 50%. The experiment uses a common pseudorandom number generator to simulate the generation of CSI and obtains the initial key seeds. Subsequently, the key generation is repeated 10,000 times, and the number of times each key bit $n_{i}$ appears as 1, where *i* is a positive integer representing the key bit positions from 1 to 128, is recorded. The probability of generating a “1” for key bit *i* can then be calculated as follows:

(26) $$P_{i}=\frac{n_{i}}{10,000}\left(1\leq n\leq 128\right).$$

**Figure 12:**
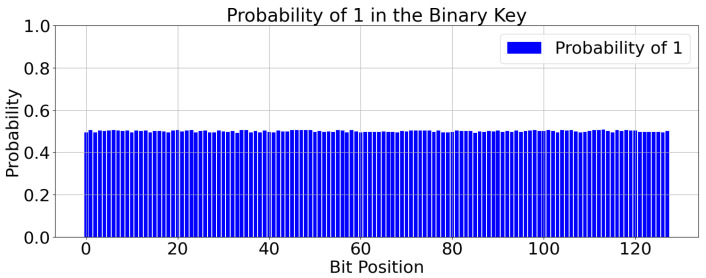
Key distribution chart.

The experimental results are shown in Figure 12: as can be seen from the diagram, the probability of generating a 1 for each key bit approaches 50%. This indicates that NDPCS-KS generates keys with randomness, and the probability of each bit being 0 or 1 approaches 50%, making it difficult to predict.

#### 6.4.2. Monte Carlo Simulations

Next, the analysis explains why NDPCS-KS ensures that the probability of each key bit being 0 or 1 approaches 50%. NDPCS-KS includes three steps:

1. Random generation of base_point: 25 base_point are randomly generated within the continuous two-dimensional space [0, 10] × [0, 10].
2. Random generation of negotiation_value: A point is randomly generated within the same two-dimensional interval, and whether this point falls within the neighborhood of any base_point is determined.
3. Consideration of overlapping neighborhoods: The neighborhood of each base_point may overlap with other base_point, which increases the complexity of calculating the probability that a single point falls within a neighborhood.

Due to the involvement of continuous two-dimensional random variables and overlapping areas, precise calculation using traditional probability methods is too complex and may lack a closed-form solution.

Therefore, the Monte Carlo method is utilized to estimate the probability that a random point falls within the neighborhood of at least one base_point by generating many random samples. We calculate the probability that a random point, uniformly distributed in the range [0, 10] × [0, 10], falls within the neighborhood (R=1.5) of at least one of 25 base_point also defined within the same range. The specific simulation steps are as follows:

1. Generate base_point: Randomly generate 25 base_point in the two-dimensional interval [0, 10] × [0, 10].
2. Generate random points: Generate many random points within the same range.
3. Calculate neighborhood hits: For each random point, check whether it falls within the neighborhood of any base_point.
4. Estimate probability: Calculate the proportion of random points within at least one neighborhood.

**Figure 13:**
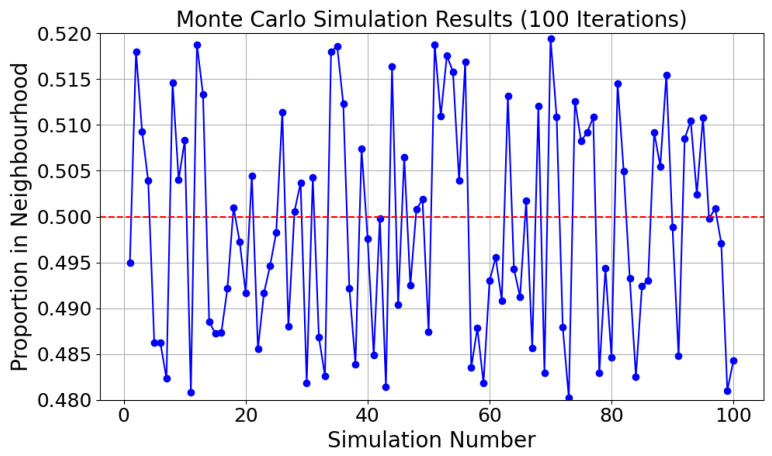
Monte Carlo simulation results.

The probabilities obtained from 100 rounds of Monte Carlo simulations are calculated, and the results are shown in Figure 13.

According to Figure 13, most of the estimated probabilities are around 50%, which also reflects the randomness of NDPCS-KS.

#### 6.4.3. Entropy Statistics

Additionally, the key entropy can also reflect its randomness. Specifically, entropy is calculated by determining the frequency and distribution of 0s and 1s in the key. The equation for calculating entropy involves the probabilities of each element (here, 0 and 1), which are used to calculate the average information content of the entire key. The calculation of entropy is as follows:

(27) $$H\left(X\right)=-\sum_{i=1}^{n}P\left(x_{i}\right)\log_{2}P\left(x_{i}\right).$$

$P(x_{i})$ is the probability of the random variable taking a specific value $x_{i}$, and *n* is the total number of possible values of *X*.

**Figure 14:**
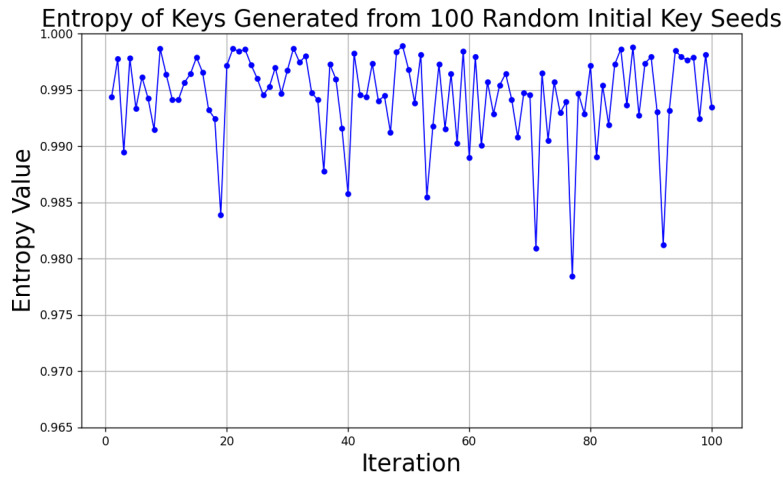
Entropy statistics of keys.

The closer the results are to the theoretical maximum value (1 for binary keys), the more it indicates that each bit of the key is nearly equally likely to be 0 or 1, ensuring satisfactory randomness and security. The key generation process is repeated 100 times, and the results are shown in Figure 14: the entropy values of the keys generated by NDPCS-KS all approach 1, indicating that the keys have excellent randomness and a high degree of uncertainty. Such keys are difficult to predict, therefore enhancing their security.

#### 6.4.4. NIST Statistical Test Suite

Additional results of key randomness testing are shown in Table 3 below, which shows the results of testing NDPCS-KS in the NIST Statistical Test Suite [42]. The NIST Statistical Test Suite, developed by the National Institute of Standards and Technology (NIST), is a collection of tests designed to evaluate the randomness and statistical properties of random number generators. It includes tests such as the frequency test, block frequency test, cumulative sums test, runs test, longest run test, matrix rank test, discrete Fourier transform (FFT) test, template matching test, universal statistical test, approximate entropy test, and linear complexity test. These tests help ensure that random number generators produce sequences with the expected statistical characteristics, making them essential for applications in cryptography, secure communications, and other fields requiring high levels of randomness.

A length of 10,000,000 bits of 0s and 1s was generated for testing with the bitstream set to 10. The minimum pass rate for each statistical test, with the exception of the random excursion (variant) test, is approximately 8 for a sample size of 10 binary sequences. For the random excursion (variant) test, the minimum pass rate is approximately 3 for a sample size of 4 binary sequences. The standard for passing the tests is a *p*-value greater than 0.01. As shown in Table 3, the experimental results indicate that the random sequences generated by the proposed algorithm passed all randomness tests, validating the robustness and reliability of the generated keys in terms of randomness, making them suitable for applications requiring high security.

## 7. Conclusions

In the paper, we proposed a key synchronization method, NDPCS-KS, based on NDBs and physical channel state characteristics in WSNs. Due to the increasing number of network nodes, centralized CA/PKI authorities face challenges in distributing, updating, and managing keys and are vulnerable to attacks. To achieve key distribution without CA/PKI or other trusted third-party facilities, a novel key synchronization method based on negative databases and physical channel state characteristics of WSNs is designed. The communicating parties obtain the initial key seeds through the initial negotiation. The negotiation keys and NDBs are constructed using the initial key seeds. The NDBs are then used for the second negotiation of the sequence to obtain the final communication keys. The key synchronization process involves a one-way hash function, isolation of the physical channels and the NP-hard characteristic of reversing the NDB. It is computationally infeasible for attackers to deduce the key seeds that produced the NDBs using any eavesdropped data. Furthermore, the second negotiation process based on the NDBs makes it impossible for the attackers to guess the legitimate communication keys. Additionally, an identity verification mechanism and a lightweight anti-replay mechanism are introduced to prevent replay, forgery, and tampering attacks in WSNs.

The theoretical analysis demonstrated that attackers cannot obtain legitimate communication keys without the initial key seeds. Experimental results proved that NDPCS-KS effectively counters replay, tampering, and forgery attacks, while its key distribution is more efficient than other solutions. Lastly, the randomness of NDPCS-KS is also verified.

## Figures and Tables

**Table 1:** Schematic diagram of an NDB.

DB	U-DB	NDB
	001	
000	010	0*1
111	011	*11
	100	10*
	101	
	110	

**Table 2:** Basicdefinition of the system.

Term	Definition
Main channels	The legitimate channels between any pair of sensor nodes
$K_{a\to b}$	The communication key between child node *a* and parent node *b*
$E(K_{a\to b},x)$	Message *x* encrypted with $K_{a\to b}$
$D(K_{a\to b},E\left(K_{a\to b},x\right))$	Message *x* decrypted with $K_{a\to b}$
${row\_seed}_{a\to b}$	The initial key seed is negotiated via CSI between nodes *a* and *b*, with a→b indicating *a* as the child node and *b* as the parent node
${row\_seed}_{a\to b}\left[i\right]$	Represents the *i*th bit of $row\_seed_{a\to b}$, where *a* is the child node of *b*
${row\_seed}_{a\to b}\left(t\right)$	$row\_seed_{a\to b}$ used by node *a* to communicate with node *b* at time *t*
RS_Library	Initial key seeds library maintained by each node
$base\_point=(x_{i},y_{i})$	The two-dimensional baseline points of the NDB
NDB_base	All base_point elements form an NDB_base sequence
NDB_base[i]	Represents the *i*th base_point element of NDB_base
$negotiation\_value=(x_{j}^{\prime },y_{j}^{\prime })$	The two-dimensional negotiation values
negotiation_KEY	All negotiation_value elements form the negotiation key $negotiation_{K}EY$ for NDB second negotiation
negotiation_KEY[j]	Represents the *j*th negotiation_value element of negotiation_KEY
*R*	The initial filtering radius
Δ	The adjustment value for dynamic radius adjusting
$R_{new}$	The adjusted filtering radius
filtered_key	A sequence representing the communication key
filtered_key[j]	The *j*th bit of the communication key, which can be 0 or 1
$v_{a\to b}$	Identity verification value calculated by child node *a* sending information
$v_{a\to b}^{\prime }$	Identity verification value calculated by parent node *b* receiving information
RHT_Library	A library of rolling hash tokens maintained by each sensor
Time_Stamp	A timestamp includes the generation time $T_{gen}$ and the token expiration time $T_{exp}$
TK	A token calculated by sensor *a* using SHA256 with $E(K_{a\to b},Time\_Stamp)$ as input, sent to sensor *b*

**Table 3:** Results of NIST randomness tests for NDPCS-KS.

Test Name	*p*-Value	Proportion Passing
Frequency Test	0.350485	10/10
Block Frequency Test	0.534146	9/10
Cumulative Sums Test 1	0.739918	10/10
Cumulative Sums Test 2	0.911413	10/10
Runs Test	0.213309	9/10
Longest Run Test	0.350485	10/10
Rank Test	0.350485	10/10
FFT Test	0.534146	10/10
Non-Overlapping Template Test 1	0.122325	10/10
Non-Overlapping Template Test 2	0.350485	10/10
Non-Overlapping Template Test 3	0.739918	10/10
Non-Overlapping Template Test 4	0.534146	10/10
Non-Overlapping Template Test 5	0.739918	9/10
Overlapping Template Test	0.739918	10/10
Universal Statistical Test	0.534146	10/10
Approximate Entropy Test	0.534146	10/10
Random Excursions Test 1	—	3/4
Random Excursions Test 2	—	4/4
Random Excursions Test 3	—	4/4
Serial Test 1	0.739918	10/10
Serial Test 2	0.739918	10/10
Linear Complexity Test	0.035174	10/10

## Data Availability

No new data were created or analyzed in this study.

## References

[1] Al-Fuqaha A., Guizani M., Mohammadi M., Aledhari M., Ayyash M. (2015). Internet of Things: A Survey on Enabling Technologies, Protocols, and Applications. IEEE Commun. Surv. Tutor..

[2] Shafique K., Khawaja B.A., Sabir F., Qazi S., Mustaqim M. (2020). Internet of Things (IoT) for Next-Generation Smart Systems: A Review of Current Challenges, Future Trends and Prospects for Emerging 5G-IoT Scenarios. IEEE Access.

[3] Lombardi M., Pascale F., Santaniello D. (2021). Internet of Things: A General Overview Between Architectures, Protocols and Applications. Information.

[4] Abiodun O.I., Oluwaranti A., Misra S., Chamola V. (2021). A Review on the Security of the Internet of Things: Challenges and Solutions. Wirel. Pers. Commun..

[5] Lin Y., Xie Z., Chen T., Cheng X., Wen H. (2024). Image Privacy Protection Scheme Based on High-Quality Reconstruction DCT Compression and Nonlinear Dynamics. Expert Syst. Appl..

[6] Shen S., Chen X., Duan H., Zhang J., Xiang Y. (2014). Differential Game-Based Strategies for Preventing Malware Propagation in Wireless Sensor Networks. IEEE Trans. Inf. Forensics Secur..

[7] Chen L., Ji J., Zhang Z. (2013). Wireless Network Security.

[8] Zhou L., Kang M., Chen W. (2022). Lightweight Security Transmission in Wireless Sensor Networks Through Information Hiding and Data Flipping. Sensors.

[9] Dai H., Xu H. (2010). Key Predistribution Approach in Wireless Sensor Networks Using LU Matrix. IEEE Sens. J..

[10] Kuang W. (2010). Improved Random Key Pre-Distribution Scheme for Wireless Sensor Networks. Chin. J. Sens. Actuators.

[11] Yin L., Liu Q., Liang W., Shen H., Yang Y. Secure Pairwise Key Establishment for Key Predistribution in Wireless Sensor Networks. Proceedings of the 2012 International Conference on Computer Science and Service System.

[12] Renyi S. (2011). Dynamic Nonlinear Key Distribution in Wireless Sensor Networks. J. Yanbian Univ..

[13] Chen S., Zhang H., Wang Q. (2011). Dynamic Key Management Scheme in Wireless Sensor Networks. High Performance Networking, Computing, and Communication Systems: Second International Conference ICHCC 2011, Singapore, 5–6 May 2011, Selected Papers.

[14] Yousefpoor M.S., Barati H. (2020). DSKMS: A Dynamic Smart Key Management System Based on Fuzzy Logic in Wireless Sensor Networks. Wirel. Netw..

[15] Xiong H., Li X., Gao S., Zhang J., Zhao H. (2021). Efficient Secret Key Generation Scheme of Physical Layer Security Communication in Ubiquitous Wireless Networks. IET Commun..

[16] Altun U., Emekligil M., Ozger M. (2022). Scalable Secret Key Generation for Wireless Sensor Networks. IEEE Syst. J..

[17] Wei X., Saha D. KNEW: Key Generation Using Neural Networks From Wireless Channels. Proceedings of the 2022 ACM Workshop on Wireless Security and Machine Learning.

[18] Rangarajan J., Sharma N., Gope P., Kundu S. Crypto Analysis With Modified Diffie–Hellman Key Exchange Based Sensor Node Security Improvement in Wireless Sensor Networks. Proceedings of the 2023 Third International Conference on Artificial Intelligence and Smart Energy (ICAIS).

[19] Moara-Nkwe K., Masek P., Barcelo-Ordinas J.M., De Poorter E. (2018). A Novel Physical Layer Secure Key Generation and Refreshment Scheme for Wireless Sensor Networks. IEEE Access.

[20] Ji Z., Huang H., Zhang J., Yang F., Wen H. (2022). Physical-Layer-Based Secure Communications for Static and Low-Latency Industrial Internet of Things. IEEE Internet Things J..

[21] Aldaghri N., Mahdavifar H. (2020). Physical Layer Secret Key Generation in Static Environments. IEEE Trans. Inf. Forensics Secur..

[22] Chen Y., Li X., Du X., Zhao H. (2023). Physical Layer Key Generation Scheme for MIMO System Based on Feature Fusion Autoencoder. IEEE Internet Things J..

[23] Chen Y., Li X., Du X., Zhao H. (2023). Physical Layer Secret Key Generation Based on Bidirectional Convergence Feature Learning Convolutional Network. IEEE Internet Things J..

[24] Wunder G., Nieman K., Dahmen T. Mimicking DH Key Exchange Over a Full Duplex Wireless Channel via Bisparse Blind Deconvolution. Proceedings of the 2023 6th International Conference on Advanced Communication Technologies and Networking (CommNet).

[25] Cao Z., Lin Z., Zhou X., Niu H. Eliminating Privacy Amplification in Secret Key Generation from Wireless Channels. Proceedings of the 2015 10th International Conference on Communications and Networking in China (ChinaCom).

[26] Hua Y. (2023). Generalized Channel Probing and Generalized Pre-processing for Secret Key Generation. IEEE Trans. Signal Process..

[27] Li G., Xia Y., Chen Y., Zhang Z. The Optimal Preprocessing Approach for Secret Key Generation from OFDM Channel Measurements. Proceedings of the 2016 IEEE Globecom Workshops (GC Wkshps).

[28] Li G., Zhang Z., Liu L., Xia Y., Chen Y. (2018). High-Agreement Uncorrelated Secret Key Generation Based on Principal Component Analysis Preprocessing. IEEE Trans. Commun..

[29] Wang X., Cao Z., Li G., Zhou X., Zhou J. (2016). Secret Key Extraction with Quantization Randomness Using Hadamard Matrix on QuaDRiGa Channel. Information and Communications Security: 17th International Conference, ICICS 2015, Beijing, China, 9–11 December 2015, Revised Selected Papers.

[30] Horie S., Watanabe O. Hard Instance Generation for SAT. Proceedings of the 8th International Symposium on Algorithms and Computation (ISAAC ’97).

[31] Hwang M.-S., Li L.-H. (2000). A New Remote User Authentication Scheme Using Smart Cards. IEEE Trans. Consum. Electron..

[32] Esponda F., Forrest S., Helman P. (2009). Negative Representations of Information. Int. J. Inf. Secur..

[33] Esponda F., Ackley E., Forrest S., Helman P. (2007). Protecting Data Privacy Through Hard-to-Reverse Negative Databases. Int. J. Inf. Secur..

[34] Barthel W., Hartmann A.K., Leone M., Ricci-Tersenghi F., Weigt M., Zecchina R. (2002). Hiding Solutions in Random Satisfiability Problems: A Statistical Mechanics Approach. Phys. Rev. Lett..

[35] Achlioptas D., Jia H., Moore C. (2005). Hiding Satisfying Assignments: Two Are Better Than One. J. Artif. Intell. Res..

[36] Liu R., Luo W., Wang X. A Hybrid of the Prefix Algorithm and the Q-Hidden Algorithm for Generating Single Negative Databases. Proceedings of the 2011 IEEE Symposium on Computational Intelligence in Cyber Security (CICS).

[37] Liu R., Luo W., Yue L. (2014). The P-Hidden Algorithm: Hiding Single Databases More Deeply. Immune Comput..

[38] Zhao D., Du D., Liang Y., Xu D., Ji S. A Fine-Grained Algorithm for Generating Hard-to-Reverse Negative Databases. Proceedings of the 2015 International Workshop on Artificial Immune Systems (AIS).

[39] Zhang J., Mao J., Li X., Yao W. Experimental Study on Channel Reciprocity in Wireless Key Generation. Proceedings of the 2016 IEEE 17th International Workshop on Signal Processing Advances in Wireless Communications (SPAWC).

[40] Klement F., Voss P., Koller F., Obermeier S., Parzinger M., Hollick M. Keep Your Enemies Closer: On the Minimal Distance of Adversaries When Using Channel-Based Key Extraction in SISO 6G Systems. Proceedings of the 2023 19th International Conference on Wireless and Mobile Computing, Networking and Communications (WiMob).

[41] Beaulieu R., Shors D., Smith J., Treatman-Clark S., Weeks B., Wingers L. The SIMON and SPECK Lightweight Block Ciphers. Proceedings of the 52nd Annual Design Automation Conference (DAC ’15).

[42] National Institute of Standards and Technology NIST Statistical Test Suite. https://csrc.nist.gov/publications/detail/sp/800-22/rev-1a/final.

